# Frequent attenders in late life in primary care: a systematic review of European studies

**DOI:** 10.1186/s12875-017-0700-7

**Published:** 2017-12-20

**Authors:** Franziska D. Welzel, Janine Stein, André Hajek, Hans-Helmut König, Steffi G. Riedel-Heller

**Affiliations:** 1 Institute of Social Medicine, Occupational Health and Public Health, Medical Faculty, University of Leipzig, Philipp-Rosenthal-Straße 55, 04103 Leipzig, Germany; 2 Institute of General Medicine, University of Leipzig, Leipzig, Germany; 3 Department of Health Economics and Health Services Research, University Medical Centre Hamburg-Eppendorf, Hamburg, Germany

**Keywords:** Primary care, General practice, Elderly, Frequent attendance

## Abstract

**Background:**

High utilization of health care services is a costly phenomenon commonly observed in primary care practices. However, while frequent attendance in primary care has been broadly studied across age groups, aspects of high utilization by elderly patients have not been investigated in detail. The aim of this paper is to provide a systematic review of frequent attendance in primary care among elderly people.

**Methods:**

We searched five databases (PubMed, PsycINFO, Web of Science, PubPsych, and Cochrane Library) for published papers addressing frequent attendance in primary health care among elderly individuals. Quality of studies was assessed using established criteria for evaluating methodological quality.

**Results:**

Ten studies met inclusion criteria and were included for detailed analysis. The average number of patients frequently utilizing primary care services varied across studies from 10% to 33% of the elderly samples and subsamples. The definition of frequent attendance across studies differed substantially. The most consistent associations between frequent attendance and old age were found for presence and severity of physical illness. Results on mental disorders and frequent attendance were heterogeneous. Only a few studies have assessed frequent attendance in association with factors such as drug use, social support or sociodemographic aspects; however results were inconsistent.

**Conclusions:**

Severe ill health and the need for treatment serve as the main drivers of frequent attendance in older adults. As results were scarce and divergent, future studies are needed to provide more information on this topic. Since prior studies have offered only a snapshot of this service use behaviour, a longitudinal approach would be preferable in the future.

**Electronic supplementary material:**

The online version of this article (10.1186/s12875-017-0700-7) contains supplementary material, which is available to authorized users.

## Background

General practitioners (GPs) are usually the first point of contact for elderly people for a broad range of health problems. However, a small proportion of patients, visit their GPs more frequently, thereby generating a significant amount of their workload [1, 2]. In general terms, a patient who attends general health care practices on a regular basis and who exceeds a certain number of visits within a given time interval is defined as a frequent attender (FA) [3–5]. As such, FAs are patients who consume large amounts of GP resources and generate sizable expenses for health care services [6–8]. Primary care is a main base of generalist care and serves as a major access point to the first level of professional care for people of all age groups including elderly people with depression and the oldest-old [9–12]. Thus, the primary care level provides a key setting for studying high utilization by elderly patients.

Across countries and differing definitions of frequent attendance, elderly people are overrepresented among this group of primary care utilizers [13–16]. Due to increasing life expectancy and low birth rates, the proportion of people aged 65 years and over has increased in Europe over the last decade from 16.6% in 2005 to 19.2% in 2016 [17]. In an aging society, high utilization of primary health care services among the elderly will have considerable consequences in terms of expenditure and costs for health care resources [7, 18].

While several studies have linked frequent attendance of adults of all ages to chronic physical illness [19–22], mental disorders [19, 21, 22] and female gender [16, 21, 23], the increased utilization of health care services by the elderly has also been associated with depressive symptoms, migration status, lower income and lower educational levels [24–27]. As elderly people are often more vulnerable to a variety of illnesses and life stressors, it may be reasonable to assume that older FAs have different reasons for frequent attendance than their younger counterparts.

Previous studies [28, 29] have reviewed frequent attendance in general practice in all age groups; however, they did not specifically look at the frequent attendance of elderly people in detail. To date, an overview of frequent attendance by the elderly that provides information on the associations and determinants of elderly FAs in primary health care is lacking. Furthermore, an understanding of the factors associated with frequent attendance among the elderly is important for planning for and providing cost-effective and target-oriented health care. This review sets out to fill this gap by reviewing relevant literature on frequent attendance among the elderly at the primary care level.

Unfortunately, cross-national comparisons of health care related issues are challenging because of the differences in health care systems and conflicting definitions of primary care across countries. Although health care is mainly a national matter, even in the EU, there is some agreement among European countries about the importance and role of general practitioners in delivering primary health care services [30, 31]. Therefore, this review focuses on European studies.

The objectives of this review are: (1) to systematically obtain and evaluate the relevant literature on frequent attenders in primary health care among the elderly in Europe, (2) to provide an overview and information source about elderly people frequently attending primary health care practices, and (3) to discuss the potential determinants of frequent utilization of primary health care services in old age.

## Methods

This review follows the PRISMA (Preferred Reporting Items for Systematic Reviews and Meta-Analyses) guidelines [32].

### Search terms and search strategy

A systematic literature search in the electronic databases PubMed, PsycINFO, Web of Science, PubPsych and Cochrane Library was conducted in November 2016. No restriction regarding the year of publication was imposed. Electronic databases were searched using MeSH keywords and free-text terms as follows: (high utiliz* OR heavy use* OR (frequent AND (consult* OR attend* OR use*))) AND (“Physicians, Primary Care” OR “Physicians, Family” OR “General Practitioners” OR “Primary Health Care” OR “Family Practice”) AND (old age OR elderly). For full search strategy see Additional file 1.

### Selection criteria

Abstracts were screened using the following selection criteria: (i) published studies in the primary care/general practice settings, (ii) patient recruitment in primary care/general practice, (iii) study samples consisting of patients aged 65 years and older, (iv) studies focused on frequent attendance, (v) measurement of frequency by number of contacts with general practice, (vi) studies providing an explicit definition of frequent attendance, (vii) studies conducted in Europe. Criteria for exclusion were as follows: (viii) language other than English or German, (ix) literature review only, (x) no full report of primary research (e.g. conference abstract, commentaries, study protocol), (xi) single case studies, (xii) setting is not exclusively general practice, (xiii) studies assessing primarily other aspects of health care consultation than frequent attendance.

### Data extraction and data synthesis

Abstracts and titles were screened and potentially relevant articles were retrieved in full-text for a more detailed analysis. Duplicates were eliminated. In addition, the bibliographies of selected articles were assessed for further relevant literature. Identified studies that were likely to be relevant were assessed in full-text according to the above described selection criteria. Data extraction was conducted using predetermined criteria based on study characteristics and main results. The following information was extracted from each included study: a) study characteristics (authors, year, country, study design and objectives), b) sampling and characteristics of participants (study base, sampling from study base, sample gender and age, control sampling, number of participating GPs), c) definition of frequent attendance including contact initiation, included and excluded contacts, data sources and d) main results for the elderly, median or mean consultation rate and odds ratio for frequent attendance, if provided. A narrative synthesis approach was applied to describe key associations of frequent attendance in old age.

### Quality assessment

The methodological quality of the studies included in this review was independently evaluated by the principal author (F.W.) and co-author (J.S.) using a 13-item checklist of predefined criteria. The checklist (see Table 1) was build based on established criteria lists applied in other reviews [33–36]*.* As not all of the 13 criteria were applicable to all 10 studies due to differing study designs, the overall quality of a study was assessed using the number of applicable checklist items as reference value. Studies scoring in the 75th percentile or higher were rated as high quality, while studies scoring between 50% and 75% were rated as moderate quality, and studies scoring below 50% were categorized as low quality.

**Table 1:** Criteria for assessing methodical quality of studies on frequent utilization of primary health care services

Study objective and design	
1.	Clearly stated study objectives and hypotheses.
Study population	
2.	Study sample is nationally and regionally representative, study sample includes representative sample of elderly individuals.
3.	Sample inclusion and/or exclusion criteria are formulated.
4.	Sociodemographic characteristics of the study sample are described.
5.	Participation and response rates are reported, Participation rate > 75%.
Assessment	
6.	Detailed description of methods, procedures and instruments is given.
7.	Stratification (e.g. age, gender) was used to assess frequent attendance.
Data reporting and analysis	
8.	Characteristics of responders and non-responders are presented.
9.	Descriptive data (mean or median, standard deviations or percentages) are provided for the most important outcome measures and for different age groups.
10.	Data on frequent attendance among elderly is given.
11.	Precision of estimates is given (e.g. 95% Confidence Intervals).
12.	The handling of missing values is described.
Other	
13.	Conflicts of interest reported and identification of funding sources is possible.

Adapted from [33–36]

Each item is scored as 1 = met the quality criterion, 0 = did not meet the quality criterion or item was not reported or unclear, − not applicable

## Results

### Literature search results

**Fig. 1:**
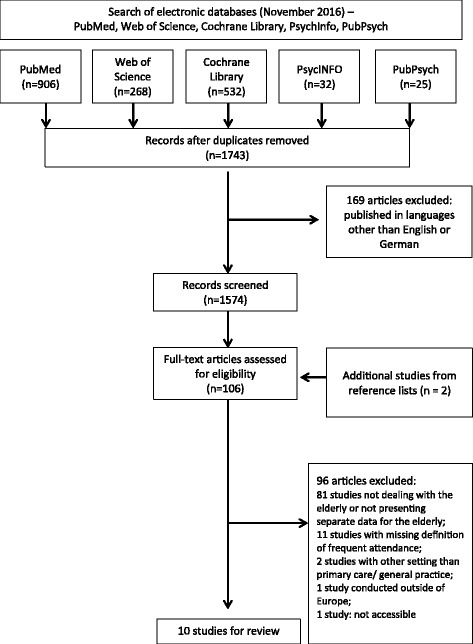
PRISMA flowchart showing the different phases of the selection process

The initial search strategy yielded 1743 potentially relevant articles, of which 169 studies were excluded as they were published in languages other than English or German. For 1574 articles, titles and abstracts were screened for eligibility. From these, 104 studies were identified as eligible and two additional articles were chosen from the bibliographies of other articles. Overall, 106 studies were obtained and reviewed for final inclusion. After full-text assessment, 10 studies were identified and considered for detailed analysis. The different stages of this selection process are provided in Fig. 1.

### Methodological quality

The results of the methodological quality assessment of the included ten studies are displayed in Table 2, right column. Six studies (60%) were of high quality, two studies (20%) of moderate quality, and two studies (20%) of low quality. The median score per study was nine (range five to ten) for cross-sectional and cohort studies. Methodological shortcomings according to the quality assessment (items with a score below the median) were lack of representativeness of the sample, lack of stratification for assessment of frequent attendance, missing reports on sociodemographic details and insufficient report or handling of missing values.

**Table 2:** Study characteristics and study quality of the included studies

Author and year	Country	Study type	Study population	Sample			Number GP’s/practices	Study quality (score)
				n (FA)/n (Controls)	Age	Sex		
Bergh and Marklund, 2003 [37]	Sweden	cross-sectional	listed patients	elderly (≥65 years): 85/126	women (≥65 years): M = 77.1 (Fa), M = 76.4 (CG) men (≥65 years): M = 77.0 (Fa), M = 74.1 (CG)	elderly (≥65 years): 46.4% female	7/1	High quality (9)
Gilleard et al., 1998 [38]	UK	cross-sectional	listed patients aged 65 years and over	95/919	range: ≥65	n/a	n/a /1	Low quality (6)
Menchetti et al., 2006 [39]	Italy	cross-sectional	attending patients aged 14 years and older	elderly (≥ 60 years): 136/470	elderly (≥ 60 years): n/a	elderly (≥ 60 years): 61% female	191/ n/a	High quality (10)
Rennemark et al., 2009 [40]	Sweden	cross-sectional	registered patients with the Swedish National Study on Aging and Care	229/511	Median = 66 range: 60–78	54.2% female	n/a	High quality (10)
Scherer et al., 2008 [46]	Germany	cohort-study	listed patients with diagnosis of heart failure	48/262	M = 72.9 (SD = 9)	68.8% female (FA)/ 50.4% female (CG)	n/a /44	Moderate quality (8)
Sheehan et al., 2003 [45]	UK	cross-sectional	attending patients aged 65 years and over	53/87	M = 76.8 (SD = 7.3) range: ≥ 65	57.9% female	14/2 (centres)	Moderate quality (9)
Svab and Zaletel-Kragelj, 1993 [43]	Slovenia	cross-sectional	listed patients	elderly (>65): 34/78	elderly (>65): n/a	elderly (>65): n/a	8/1 (primary care centre)	Low quality (5)
van den Bussche et al., 2016 [44]	Germany	cross-sectional	registered patients with a health insurance company	23,590 (19.1%)/99,634 (80.9%)	FA: M = 73, SD = 6.4 CG: M = 71.7, SD = 6.1	46.3% female (FA)/ 41.4% female (CG)	n/a	High quality (9)
Vedsted et al., 2001 [42]	Denmark	cross-sectional	listed patients aged 20 years and over	elderly (>65): n/a	elderly (>65): n/a	elderly (>65): n/a	n/a	High quality (9)
Vedsted et al., 2004 [41]	Denmark	cross-sectional	attending patients aged 20 years and over	elderly (≥65 years):6718/n/a	elderly (≥65 years): n/a	elderly (≥65 years): 57.8% female (FA)	320/179	High quality (10)

*FA* Frequent attender, *GP* General Practitioner, *M* Mean, *SD* Standard deviation, *CG* Control group; n/a = no information provided; − = not applicable

### General and methodological characteristics of the reviewed studies

Methodological characteristics of the reviewed studies and results regarding frequent attendance in elderly samples are summarized in Tables 2 and 3.

The included studies came from the following European countries: the United Kingdom, Sweden, Germany, Denmark (two studies each), as well as Italy and Slovenia (one study each).

Most of the included studies in this review applied a cross-sectional design [37–45]. One study reported the use of a longitudinal design [46]; however, the authors did not apply a specific regression model to analyse the panel data but instead used baseline data to predict subsequent frequent attendance.

The samples of the majority of the reviewed studies were based on the population of listed patients from cooperating GPs, primary health care centres or patients registered with a health insurance company [37, 38, 42–44]. In one study, the study population consisted of listed patients with the documented diagnosis of heart failure [46]. In three out of the ten studies, the study population consisted of patients attending a general practice or primary health care centre during a predefined time interval [39, 41, 45]. In one Swedish study, the study sample was based on patients registered with the Swedish National Study on Aging and Care [40]. Only half of the included studies focused solely on elderly patients [38, 40, 44–46]. Half of the studies assessed frequency of attendance in subsamples of elderly patients among other age groups [37, 39, 41–43].

Sample sizes considering elderly samples or subsamples varied substantially across studies between *n* = 112 and *n* = 123,224. Two studies did not provide detailed information on the subsamples of elderly patients [41, 42]. Seven studies included information on gender distribution reporting a proportion of 41.4% to 68.8% female patients [37, 39–41, 44–46], with only two studies reporting a larger proportion of male patients [37, 44].

The number of participating GPs differed substantially across the included studies between *n* = 7 and *n* = 320; the number of practices varied between n = 1 and *n* = 179. However, three studies failed to report any information on the number of included GPs and practices [40, 42, 44], while another three studies solely reported the number of included GPs [39] or the number of included practices [38, 46].

### Definition of frequent attendance

The various definitions of frequent attendance are provided in Table 3. An overview of the percentages of FAs and thresholds for frequent attendance across studies is summarized in Table 4. The percentage of elderly patients frequently utilizing primary care services varied across studies from 10% to 33%. The majority of studies considered in this review used a proportional approach to define frequent attendance and distinguished high utilizers of primary care services from other attenders. Only three studies applied an absolute number of consultations within a specified time interval as a cut-off value between FAs and non-frequent attenders (non-FAs) [39, 44, 46]. Additionally, one of these studies considered further definitions of frequent attendance [44]. In addition to the number of consultations with primary care practices within a year, the authors applied contacts with a certain number of different practices and contacts with different practices of the same medical specialty as further definitions of FAs. Four studies allocated the top 10% of most frequent primary care users into the group of FAs [37, 38, 41, 42]. Other studies used different cut-off points to define FAs: frequencies of top 25% [43], 30% [40] and 33% [45] were each employed. While only four studies assessed attendance rates stratified by sex or age [37, 41–43], the majority of the included studies assessed attendance over a 12 month timeframe [37, 38, 40–44]. Other time intervals used to assess frequency of attendance were 6 months [39] and 9 months [45, 46].

**Table 3:** Results on frequent attendance among the elderly at the primary care level across reviewed studies

Author and year	FA definition	Included contacts	Excluded contacts	Data sources	Main results
Bergh and Marklund, 2003 [37]	10% most frequent attenders in 12 months/ by sex and age group	face-to-face visits to GP		medical records	Elderly (≥ 65 years): • Most diagnostic groups and medical prescriptions more frequent among FAs than non-FAs for both sexes Most common diseases: • Women: diseases of circulatory and musculoskeletal system, similar for FAs and non-FAs • Men: circulatory & endocrine diagnoses (FAs), circulatory and musculoskeletal problems (non-FAs)
Gilleard et al., 1998 [38]	Very High Attenders: 10% most frequent attenders in 12 months (> 15 contacts in 12 months)	face-to-face visits to GP, visits to the practice nurse	home visits, out-of-hour visits	computerized records, interviews, questionnaires	Elderly (≥ 65 years): • 10% FAs responsible for 33% of all visits • Frequent attendance not associated with psychiatric morbidity, self-reported depression, use of hypnotic or antipsychotic medication • Use of antidepressants: 9.5% of FAs received prescriptions for antidepressants compared to 2.8% of low average attenders (chi-square = 13.6, df 3, *p* < 0.01)
Menchetti et al., 2006 [39]	> 1 contact to GP per month in 6 months	n/a	n/a	registered data, questionnaires, clinical judgments of GPs	Elderly (≥ 60 years): • Frequent attendance associated with moderate or severe physical illness (aOR = 2.89, 95% CI: 1.63–5.11), depression (aOR = 1.92, 95% CI: 1.10–3.35) and unexplained somatic symptoms (aOR = 1.99, 95% CI: 1.05–3.77) • Depression increased risk of being an FA fivefold and was a risk factor for frequent attendance independent of other clinical predictors
Rennemark et al., 2009 [40]	30% most frequent attenders in 12 months (≥3 contacts in 12 months)	n/a	n/a	questionnaires, cognitive tests, medical records	Elderly (≥ 60 years): • Number of GP visits positively correlated with age (0.53, *p* < 0.001), and comorbidity (0.93, *p* < 0.001), and negatively correlated with functional ability (−0.18, *p* < 0.001), education level (−0.12, p < 0.01) and internal locus of control (−0.12, p < 0.01) Results from logistic regression analyses: • Physical comorbidity as main factor determining frequent attendance (OR = 8.17, 95% CI: 5.54–12.04) • Sense of coherence (OR = 1.03, 95% CI: 1.00–1.06) and locus of control (OR = 1.14, 95% CI: 1.02–1.27) significantly related to frequent attendance • Education level and social anchorage not associated with frequent attendance
Scherer et al., 2008 [46]	> 17 contacts in 9 months	n/a	n/a	questionnaires, telephone interviews	Elderly: • Frequent attendance associated with female sex, living alone, severity of heart failure, psychological distress and quality of life • In multivariate analysis physical problems (OR = 1.1, 95% CI: 1.0–1.1, p < 0.001) and living alone (OR = 2.4, 95% CI: 1.1–5.1) independently related to frequent attendance
Sheehan et al., 2003 [45]	top third of attenders in 9 months	medical contacts with GP at primary care centre or at home	consultations with practice nurse	patient interview, GP records, GP assessment of patients tendency to somatise	Elderly (≥ 65 years): • Frequent attendance related to depression (OR = 2.24, 95% CI: 1.11–4.50, *p* < 0.05), high rates of physical disorder (OR = 1.78, 95% CI:1.16–2.71, p < 0.05), somatic symptom reporting (OR = 1.83, 95% CI:1.13–2.97, p < 0.05), and low social support (OR = 1.73, 95% CI:1.01–2.94, p < 0.05) • In multivariate regression only low social support and somatic symptoms significantly related to frequent attendance
Svab and Zaletel-Kragelj, 1993 [43]	25% most frequent attenders in 12 months/ by age group	face-to-face visits with GP, contacts for administrative purposes	telephone contacts	medical records and registered data	Elderly (>65): • Probability for superficial (administrative) contacts larger for FAs compared to non-FAs (median percentage of superficial contacts among all contacts: FAs = 27.1%/non-FAs = 0.5%, *p* = 0.05) • Non-significant trend: larger probability of referral to specialists for FAs compared to non-FAs (median index-value for referral to a specialist: FAs = 8.0/non-FAs = 0.4).
van den Bussche et al., 2016 [44]	A: ≥ 50 contacts with physician practices in 12 months B: contacts with ≥10 different practices in 12 months C: contacts with ≥3 different practices of the same medical specialty in 12 months	visits to the practice, home, nursing home visits, telephone contacts, contacts with practice staff	appointments by phone and administrative contacts	insurance claims data/ registered data	Elderly (≥65): • Type A attendance associated with higher age, dependency on nursing care, multi-morbidity, and high impact somatic diseases • Types B and C attendance associated with younger age, less dependency on nursing care, and presence of mental diseases • Number of chronic conditions reduced the risk of being Type C FA
Vedsted et al., 2001 [42]	daytime: 10% most frequent attenders (≥ 12 contacts) in 12 months/ by sex and age group out-of-hours: 10% most frequent attenders (≥ 4 contacts) in 12 months	daytime: face-to-face visits with GP out-of-hours: telephone advice, surgery consultations, home visits	telephone contacts during daytime and administrative and routine consultations	electronic records	Elderly (≥ 65 years): • Frequent attendance during daytime strongly related to the risk of being an out-of-hours FA: OR and 95% CI of daytime users to be an FA in out-of-hours service compared to non-attenders: men with 10% most daytime contacts: OR = 72.5 (CI: 48.7–107.9) women with 10% most daytime contacts: OR = 40.7 (CI: 28.2–58.8)
Vedsted et al., 2004 [41]	10% most frequent attenders (≥ 12 contacts) in 12 months/ by sex and age group	face-to-face visits to GP, home visits during daytime	telephone contacts, administrative and routine consultations (e.g. driver’s licenses)	electronic records	Elderly (≥ 65 years): • Prevalence ratio for using one or more drugs only slightly higher among FAs compared to the 50%-group with the fewest contacts • Prevalence for polypharmacy (drugs from 5 or more drug groups) 6.7 times (men) and 4.2 times (women) higher among FAs compared to the 50%-group with the fewest contacts

*n/a* No information provided, *FA* Frequent attender, *fa* Frequent attendance, *GP* General practitioner, *OR* Odds ratio, *aOR* Adjusted Odds ratio, *CI* Confidence interval, *df* Degrees of freedom, *p p*-value

**Table 4:** Overview of frequent attendance in elderly samples or sub-samples across included studies

Author and year	Percentage of FAs	Threshold for frequent attendance
Bergh and Marklund, 2003	10%	n/a
Gilleard et al., 1998	10%	> 15 contacts in 12 months
Menchetti et al., 2006	22.4%	> 1 contact per month in 6 months
Rennemark et al., 2009	30%	≥ 3 contacts in 12 months
Scherer et al., 2008	15.5%	> 17 contacts in 9 months
Sheehan et al., 2003	33.3%	≥ 11 contacts in 12 months
Svab and Zaletel-Kragelj, 1993	25%	n/a
van den Bussche et al., 2016	In total: 19% Def. A: 14.2% Def. B: 8.9% Def. C: 5.1%	Def. A: ≥ 50 contacts in 12 months
Vedsted et al., 2001	10%	≥ 12 contacts in 12 months
Vedsted et al., 2004	10%	≥ 12 contacts in 12 months

*n/a* = No information provided; *FA* = Frequent attender; *Def*. = Definition

Half of the studies exclusively considered face-to-face contacts with a GP to assess frequency of attendance [37, 41–43, 45], while two studies included patient contacts with practice staff in general [38, 44]. The remaining studies provided no information about the nature of contacts included in the assessment of frequent attendance.

To measure frequent attendance and associated factors, nine out of the ten studies used medical records or electronic registered data as data sources [37–45]. One study relied solely on patient interviews and patient questionnaires as data sources on frequency of attendance [46].

### Factors associated with frequent attendance

Findings on factors associated with frequent attendance among elderly primary care patients are summarized in Table 5. Reported factors included: sociodemographic aspects, physical and mental illnesses, medical prescriptions, social support and different types of health care utilization.

**Table 5:** Findings on factors associated with frequent attendance among elderly primary care patients

	Bergh and Marklund, 2003	Gilleard et al., 1998	Menchetti et al., 2006	Rennemark et al., 2009	Scherer et al., 2008	Sheehan et al., 2003	Svab and Zaletel-Kragelj, 1993	van den Bussche et al., 2016	Vedsted et al., 2001	Vedsted et al., 2004
No. or severity of somatic diseases	+		+	+	+	+		+/−		
presence of mental illness/psychological distress		0	+		+	+		+		
medical prescriptions	+	0/+								+
low social support or social anchorage				0	0	+				
sociodemographic factors:										
older age				+	0	0		+/−		
female gender			0	+	+	0				
educational level			0	−/0						
living alone					+					
lower quality of life					+					
No. of superficial contacts							+			
No. of referrals to specialists							0			
frequent attendance out-of-hours									+	

A plus sign indicates a positive association between frequent attendance and the respective factor; a minus sign indicates a negative association between frequent attendance and the respective factor; 0 indicates no association was found; blank cells mean that the factor was not studied; No. = Number

### Sociodemographic factors

Four studies reported on the relation between *gender* and frequent attendance at the primary care level. Analysing the relationship between gender and attendance rate, two studies found no association between gender and FA-status [39, 45]. Two studies reported female gender to be significantly associated with frequent attendance [40, 46].

Assessing *age* and frequent attendance among the elderly, no significant association was reported in two studies [45, 46]. Another study found a strong positive correlation between age and number of visits to the GP (*r* = 0.53, *p* < 0.01), however this association disappeared after logistic regression analysis [40]. One further study reported odds ratios of age and gender for the relative chance of belonging to three different types of frequent attendance [44]. They found significantly higher chances for older male and female patients (≥ 75 years) of belonging to the group of FAs consulting 50 times or more within 12 months (females: OR = 1.55, 95% CI: 1.40–1.72; males: OR = 1.38, 95% CI: 1.26–1.51) compared to somewhat younger patients (65–74 years). The authors further found that the chances of belonging to the group of FAs who contacted more than nine different practices (females: OR = 0.65, 95% CI: 0.60–0.71; males: OR = 0.79, 95% CI: 0.73–0.85) or more than two practices of the same medical specialty (females: OR = 0.60, 95% CI: 0.55–0.66; males: OR = 0.88, 95% CI: 0.81–0.95) within one year was smaller in the older age groups compared to younger patients (65–74 years) [44].

Similar to age and gender, only two studies reported results on the association between *educational level* and FA-status. One study reported a significant negative correlation (*r* = −0.12, *p* < 0.01) between educational level and number of GP visits, indicating that a higher educational level was associated with a lower number of visits to the GP. However, educational level was not found to be related to frequent attendance in logistic regression analysis [40]. Another study found no association between educational level and FA-status among the elderly [39]. One further study looked at social class with regards to high and low primary care attenders and found no significant differences between the two attendance groups [45].

Two studies reported on *living situation* and *civil status*. One study found that living alone was a significant predictor of being an FA (OR = 2.4, 95% CI: 1.2–5.1, *p* = 0.02) [46]. Another study found no association between marital status (single, married, divorced or separated) and frequent attendance [39].

### Physical illness

Six out of the ten studies reported results on the association between frequent attendance at the primary care level and presence of physical diseases or multi-morbidity [37, 39, 40, 44–46]. Four studies analysed odds ratios to compare presence or severity of physical disorders among FAs and non-FAs. Three studies reported significantly higher odds for the presence of multiple physical disorders [40, 45] or moderate to severe physical illness and unexplained somatic complaints [39] among FAs as compared to non-FAs. One study computed odds ratios for the most common medical diagnoses separately to describe the ratio of individuals among FAs and non-FAs with a certain diagnosis [37]. Most of the diagnoses (circulatory disease, musculoskeletal disease, endocrine and respiratory disease) were found more frequently among FAs as compared to non-FAs. This pattern was similar for male and female patients. However, the types of medical problems FAs and non-FAs consulted their GP for were similar [37]. Another study focusing on patients with a preceding heart failure diagnosis found significantly more physical problems and higher levels of self-rated severity of heart failure-related impairments among FAs than non-FAs, whereas perceived low severity of heart failure among the elderly patients was associated with infrequent attendance [46]. The presence of physical problems linked to the heart failure diagnosis remained associated with frequent attendance even after multivariate analysis [46]. One study reported odds ratios for multi-morbidity and dependency on nursing care for three subtypes of frequent attendance [44]. They found that number of chronic conditions and nursing care dependency was significantly associated with the group of FAs consulting physician practices 50 times or more within 12 months (type A). However, the authors showed that dependence on nursing care lowered the chance of belonging to the group of FAs who contacted either more than two practices of the same medical specialty (not including general practice or internal medicine, type C) or more than nine different physician practices within a year (type B). As for multi-morbidity, the authors reported that every additional chronic disease raised the chance of belonging to the subgroup of type A attenders by 23%, to type B attenders by 4% and lowered the chance of belonging to type C attenders by 6% [44]. In summary, six out of the ten reviewed studies reported an association between presence and severity of medical diagnoses and frequent attendance at the primary care level.

### Mental illness

Four studies assessed the relationship between psychiatric morbidity [38, 39, 45] or psychological distress [46] and frequency of attendance at the primary care level. Two studies used self-assessment questionnaires to screen for depression [38] or anxiety and depressive mood [46]. Two further studies used a combination of self-assessment questionnaires and semi-structured interviews [45] or the GP’s clinical judgment [39] of the presence of psychiatric disorders. In three of these four studies, significant associations were found [39, 45, 46]. One study showed that psychological distress measured with the Hospital Anxiety and Depression Scale was significantly higher among FAs [46]. Two further studies found depression significantly associated with frequent attendance [39, 45]. However, in two out of the three studies reporting a significant association between psychological distress or depression and FA-status, anxiety and depression were no longer significant after multivariate analysis [45, 46]. Only one out of these four studies found psychiatric morbidity and self-reported depression not to be associated with frequent attendance [38].

One further study calculated relative risks (RR) of becoming a frequent attender based on a single diagnosis [44]. They found that patients with the diagnosis of anxiety disorder had a risk of belonging to the group of FAs that was 2.5 points higher than belonging to the group of non-FAs. Similar results were found for the diagnoses of somatoform disorders (RR: 2.33) and depression (RR: 2.30). The relative risks of anxiety disorder and somatoform disorder were highest for the subgroup of FAs who contacted more than nine different practices during one year [44].

### Medication

Medication and frequent attendance was assessed in three studies. One study showed that the prevalence of polypharmacy was 6.7 times higher among male FAs and 4.2 times higher among female FAs compared to the 50%-group of elderly patients with the fewest primary care contacts [41]. In another study, FAs were found to receive medical prescriptions for most prescribed drug groups (infection, neurological, circulatory and blood diseases) more frequently than non-FAs with odds ratios ranging from 1.3 to 5.1 for FAs and 0.5 to 1.4 for non-FAs [37]. Another study assessed the use of psychotropic drugs and attendance rates. They found no statistically significant association between the overall use of psychotropic drugs and category of attendance (low attendance, average attendance, high and very high attendance) [38]. However, they found an association between the use of antidepressant medications and the 10% most frequent attenders. Significantly more FAs (9.5%) were taking antidepressant medications than low average attenders (2.8%) (Chi-square = 13.6, *p* < 0.01) [38].

### Social support and social anchorage

Three studies reported results on the association between social anchorage [40] or perceived social support [45, 46] and frequency of attendance. While one study found no association between attendance rates and self-reported social anchorage [40], two studies on social support showed contrasting results. Scherer et al. [46] found perceived social support not to be associated with frequency of attendance, whereas Sheehan et al. [45] found that FAs had significantly higher rates of perceived low social support than non-FAs*.* However, the number of studies on social support and elderly FAs was small and the comparability of these studies may be limited due to large differences in the measures of social support and social anchorage.

### Type of health care utilization

The association between frequent attendance during regular office hours and frequent attendance during out-of-hours services at the primary care level was assessed in one study [42]. The results showed a strong risk of elderly daytime FAs to be FAs of out-of-hours services with odds ratios ranging from 40.7 (CI: 28.2–58.8) for female FAs to 72.5 (48.7–107.9) for male FAs.

Another study compared the type of health care utilization at the general practice and referral to specialists for FAs and non-FAs [43]. They found a significantly larger probability for superficial contacts (contacts for administrative purposes: e.g. repeat prescriptions) among FAs compared to non-FAs (see Table 4), whereas the difference in referral pattern was not significant.

## Discussion

The objective of this paper was to systematically review existing literature on elderly FAs at the primary care level in Europe and to provide an overview and information source about older primary care patients belonging to this group of health care utilizers. While the number of studies focusing solely on elderly FAs was small, frequent attendance among elderly patients was most consistently associated with the presence and severity of physical illness. The studies included in our evaluation were found to be mostly of high to moderate quality. Lack of sample representativeness and insufficient reporting of missing values were the most common shortcomings.

### General and methodological characteristics

#### Study Design

Cross-sectional studies on frequent attendance provide a snapshot of characteristics related to excessive health care consultations. However, previously published literature has shown that a considerably lower proportion of patients persist as FAs over a period of several years compared to the proportion of FAs identified in a shorter time slot (e.g. a calendar year) [21, 47] indicating that frequent doctor-consultations are typically a self-limiting behaviour. Furthermore, persistent FAs have been associated with more physical diseases, as well as social and psychiatric problems than short-term FAs [21]. Smits et al. [21], therefore, suggest that only the phenomenon of persistent frequent attendance should be studied in detail. Thus, while most of the reviewed studies were cross-sectional, the nature and course of a frequent health seeking behaviour may require long-term assessment to achieve a deeper understanding.

#### Countries of origin

While considerable variation of the task profiles of primary care providers have been found within and across European countries [48, 49], the countries included in this review seem to share similar trends in the development of GP service profiles over the last two decades. Schäfer et al. [50] reported a general increase in GPs participation in disease management across these countries, whereas several other task profiles of GPs (including GP as first contact, performance of medical technical procedures and preventive care) have declined in their frequency within the last twenty years in most countries with the exception of Sweden and Slovenia [50]. To our knowledge, frequent attendance has not been assessed with regard to differences at the primary care sector across European countries. Still, when studying frequent attendance at the primary care level it seems reasonable to assume that the context primary care is embedded within a country (e.g. dimensions of structure or service-delivery of primary care) may influence utilization behaviour.

#### Definition of frequent attendance

The methods used to differentiate FAs from other attenders in primary care practices varied substantially across the included studies. This resembles the results from Vedsted and Christensen [28], who could not find a widely accepted approach for the definition of frequent attendance in general practice. The way in which frequent attendance is defined may have relevant implications on the factors associated with this phenomenon [51]. The vast majority of the studies included in this review considered a proportional approach. Applying a proportion (e.g. the highest 10% or the highest 30%) of primary care patients into the group of FAs allows for comparisons across different studies, practices and regions. A proportional approach has the further advantage of allowing for stratification by sex and age. This allows researchers to compare FAs among patients with different sociodemographic characteristics, as women have been shown to consult their GP more frequently than men [14, 16, 23, 52] and older patients have been shown to consult more often than younger patients [13–16]. However, applying a certain proportion of most frequently attending patients stratified by sex or age into the group of FAs might fail to address the actual GP workload with respect to practical implications. GPs will usually have higher workloads with patients frequently attending the practice, whether or not those patients are within the 10% FAs of their specific sex and age group. Using an absolute number of consultations within a predefined time interval might, on the other hand, address all patients that occupy a certain amount of GP resources. Those patients are high utilizers of primary health care services even if they fail to fall into the group of FAs. Yet, the number of consultations applied as threshold for frequent attendance is often arbitrary chosen and differed substantially across the here included studies, thereby hampering the comparison of results on FAs across studies and countries. Still, in terms of practical implication, it may be useful to differentiate between high utilizers of primary care resources and FAs of a specific age and sex group.

### Main factors associated with frequent attendance

#### Physical illness

Similar to the results from Vedsted and Christensen [28] a positive association between the severity of physical disorders, multi-morbidity and frequent attendance was found among the included studies. Previous literature on FAs in primary care has consistently shown that the presence of poor health and somatic diseases, particularly chronic diseases, are significantly more prevalent among FAs than non-FAs [19–22, 53]. In line with those findings, the results of this review show that frequent attendance by the elderly is strongly associated with ill health and reflects the actual and justifiable needs of older primary care patients for frequent doctor consultations. For elderly patients with serious physical illnesses, frequently utilizing primary health care services may, therefore, be necessary and a sign of a well-developed health care provision. However, the high prevalence of polypharmacy among elderly FAs found by Vedsted et al. [41] may indicate a possible avenue for optimizing health care management for this specific patient group.

#### Mental illness

The findings on frequency of primary care contacts and presence of psychiatric morbidity were more heterogeneous. Still, Menchetti et al. [39] and Sheehan et al. [45] consider the detection and treatment of late-life depression as an opportunity to reduce or prevent frequent attendance in the elderly. The association of mental health problems and frequent attendance is in line with previous literature assessing health care utilization among the elderly [53] and the general population [19, 21, 22, 54]. Patients with depression have been found to utilize health care services more frequently than patients without depression [55, 56]. This association of depressive symptomatology and increased health care utilization seems particularly the case for the elderly. According to Press et al. [27], depressive symptoms among elderly primary care patients were a stronger risk factor for frequent attendance than sociodemographic variables or comorbidity. As recognition of mood disorders is rather poor within the primary care context [56, 57], improving recognition rates for mental disorders might provide a starting point to approach avoidable frequent attendance. However, the assessment of depression and psychiatric morbidity varied across the here reviewed studies, including short self-assessment questionnaires, semi-structured and computer-assisted interviews, as well as GPs assessment of patient’s psychiatric morbidity. As there are only a small number of studies on elderly FAs and mental illness, the findings should be interpreted with caution.

#### Further associations

The findings on further relationships between frequent attendance in the elderly and medical prescriptions, social support or sociodemographic factors were very limited and, therefore, do not allow valid conclusions on these issues. Future research on frequent attendance in the elderly should pay more attention to mediating aspects of this relationship beyond the mere presence of physical and mental diseases.

To sum up, while physical and psychological illnesses seem to be associated with frequent attendance in the elderly, several of the reviewed studies recommend further improvements in the health care management to deal with this health seeking behaviour. Vedsted et al. [42] suggest that an optimised care for daytime FAs may reduce their attendance both during the day and out-of-hours. Further recommendations involve improving the management of psychological illnesses in elderly patients [39, 45, 46], optimizing pharmacological therapy [41] or an improved integration of primary care and specialist care [39]. Van den Bussche et al. [44] argue that the phenomenon of frequent attendance appears to be multidimensional involving aspects of health care providers, patients and the health care system contributing to high utilization.

### Strengths and limitations of this study

The strength of this review is its exclusive focus on older adults, as they seem to be overrepresented among FAs. The attendance pattern of elderly patients represents a growing source of prospective health care expenditures in aging societies. To our knowledge, a targeted review of high utilization of primary health care services among the elderly in Europe has not been done before. A further strength of this review is its evaluation of the methodical quality of the included studies based on predefined and established criteria.

Yet, the present review has some limitations. First, we excluded literature published in languages other than English or German and limited our search to published work. Hence, relevant literature may have been missed. Second, only European studies were included in this paper so that results could be compared across countries with roughly similar task profiles of GPs and health care systems. Nevertheless, there is a considerable variation in primary health care settings within and between countries. Therefore, the present study may lack generalizability, specifically to non-European countries and regions. Furthermore, during the process of reviewing, it became evident that the number of papers on primary health care utilization among older adults was small. Therefore, studies that included specific results on frequent attendance among elderly patients that encompassed other age groups were also included in this review.

## Conclusions

To date, research on frequent attendance at the primary care level focusing on older adults is still unsatisfactory. Inconsistencies in the understanding of what constitutes frequent attendance hamper comparison across studies despite previous attempts in the literature to provide information on the validity of different definitions.

Still, this review indicates that severe ill health is associated with frequent attendance in the elderly and, therefore, points to a reasonable need for medical treatment as the main driver of frequent attendance in older adults. Frequent attendance is, therefore, neither right nor wrong, per se. However, it is indicative of a specific form of health seeking behaviour that should elicit stronger initiatives by health care providers so that the underlying needs of those patients can be understood.

Subsequent research is needed to shed more light on further mediating factors that contribute to frequent utilization of primary care services in the elderly. In particular, future studies should consider longitudinal approaches to study frequent attendance, carefully choose an FA definition and consider the context of primary care treatment within the health care system of their country.

## Additional file

Additional file 1: Search Strategy. (DOCX 14 kb)

## Abbreviations

CI: Confidence Interval
FA: Frequent Attender
GP: General Practitioner
OR: Odds Ratio
PRISMA: Preferred Reporting Items for Systematic Reviews and Meta-Analyses
r: Correlation Coefficient
RR: Relative Risks
