# Supplementary material for: Frequent attenders in late life in primary care: a systematic review of European studies
Source: BMC Fam Pract. 2017 Dec 20;18:104. doi: 10.1186/s12875-017-0700-7 (PMC5738881; doi:10.1186/s12875-017-0700-7)
Supplement: Additional file 1: — Search Strategy. (DOCX 14 kb) [file 12875_2017_700_MOESM1_ESM.docx]

**Search Strategy**

1. high utiliz*
2. heavy use*
3. frequent
4. consult*
5. attend*
6. use*
7. #3 and (#4 or #5 or #6)
8. #1 or #2 or #7
9. “Physicians, Primary Care” / all subheadings
10. “Physicians, Family” / all subheadings
11. “General Practitioners” / all subheadings
12. “Primary Health Care” / all subheadings
13. “Family Practice”/ all subheadings
14. #9 or #10 or #11 or #12 or #13
15. old age
16. elderly
17. #15 or #16
18. #8 and #14 and #17
